# The NH Alliance for Healthy Aging: Using a Collective Impact Approach to Advance Healthy Aging

**DOI:** 10.1093/geroni/igaa057.355

**Published:** 2020-12-16

**Authors:** Jennifer Rabalais, Laura Davie, Alison Rataj

**Affiliations:** 1 University of New Hampshire, Concord, New Hampshire, United States; 2 University of Massachusetts Boston, Boston, Massachusetts, United States

## Abstract

The NH Alliance for Healthy Aging (NH AHA) is a statewide coalition of stakeholders building cross sector partnerships that support and promote healthy aging throughout the state. Formed in 2016, NH AHA works to promote its shared vision to create communities in New Hampshire that advance culture, policies and services which support older adults and their families. As the largest coalition focused on the health and well being of older people in the state, NH AHA currently engages over 300 participants representing more than 185 organizations and/or groups statewide. Participants will hear how this collective power has led to early successes in advancing AHA’s five strategic priorities, including early statewide policy successes. A review of the collective impact model will be provided with discussion on how NH uses this model intentionally to align the work in the aging field of NH. NH AHA’s theory of change starts with changing the conversation around aging across NH’s communities and is the foundation for the efforts to advance Reframing Aging principles and recommendations through the work of NH AHA and statewide. NH AHA’s strategic plans to become a resource and hub of Reframing Aging activity in the state will be reviewed. As a result of the presentation audience members will be able to 1) describe the 5 conditions of collective impact 2) give 2 examples of how NH is utilizing these conditions intentionally and 3) describe NH’s efforts to create common measures for the aging field in NH.

